# Population Genetic Analysis of Six Chinese Indigenous Pig Meta-Populations Based on Geographically Isolated Regions

**DOI:** 10.3390/ani13081396

**Published:** 2023-04-18

**Authors:** Lige Zhang, Songyuan Zhang, Fengting Zhan, Mingkun Song, Peng Shang, Fangxian Zhu, Jiang Li, Feng Yang, Xiuling Li, Ruimin Qiao, Xuelei Han, Xinjian Li, Gang Liu, Kejun Wang

**Affiliations:** 1 College of Animal Science and Technology, Henan Agricultural University, Zhengzhou 450002, China; 2 Animal Science College, Tibet Agriculture and Animal Husbandry University, Linzhi 860000, China; 3 National Animal Husbandry Service, Beijing 100193, China; 4 National Supercomputing Center in Zhengzhou, Zhengzhou 450001, China

**Keywords:** pig, SNPs, 60K BeadChip, signature of selection, meta-populations

## Abstract

**Simple Summary:**

Whole-genome SNP data from 613 Chinese indigenous pigs were collected to comprehensively estimate the genetic diversity, genetic relationship, and population genetic structure of the indigenous pig meta-populations. These Chinese meta-populations were characterized by genetic distance, genetic difference index, and runs of homozygosity (ROH). The contribution of genetic diversity of each meta-population was scaled based on genetic and allelic diversity, which provides a basis for improving the conservation project. Further, a selective sweep analysis revealed that genes related to fat deposition and heat stress may contribute to the adaptability of the indigenous pigs to cold and heat. These findings provide a theoretical basis for the improvement of conservation strategies and insights into the environmental adaptability of Chinese indigenous pigs.

**Abstract:**

The diversification of indigenous pig breeds in China has resulted from multiple climate, topographic, and human cultural influences. The numerous indigenous pig breeds can be geographically divided into six meta-populations; however, their genetic relationships, contributions to genetic diversity, and genetic signatures remain unclear. Whole-genome SNP data for 613 indigenous pigs from the six Chinese meta-populations were obtained and analyzed. Population genetic analyses confirmed significant genetic differentiation and a moderate mixture among the Chinese indigenous pig meta-populations. The North China (NC) meta-population had the largest contribution to genetic and allelic diversity. Evidence from selective sweep signatures revealed that genes related to fat deposition and heat stress response (*EPAS1*, *NFE2L2*, *VPS13A*, *SPRY1*, *PLA2G4A*, and *UBE3D*) were potentially involved in adaptations to cold and heat. These findings from population genetic analyses provide a better understanding of indigenous pig characteristics in different environments and a theoretical basis for future work on the conservation and breeding of Chinese indigenous pigs.

## 1. Introduction

The domestication of indigenous pigs in China began about 9000 years ago [1,2,3,4], and selective breeding of pigs has been performed since the 7th century [5]. Because China is an expansive country with complex climates and geographies, domesticated breeds of pigs have experienced various degrees of natural selection leading to diverse phenotypic characteristics of indigenous pig breeds [6]. Subsequently, Chinese indigenous pig breeds have distinctive features in different geographical settings, displaying broad adaptability to multiple environments (e.g., hypoxia, hypobaria, cold, and heat) [4]. 

Based on the region where they live and environmental adaptations, Chinese indigenous pigs can be divided into six geographic types [7]. These six geographic types include Southern China (SC), Southwestern China (SWC), Central China (CC), Plateau of China (PC), Jianghai district of China (JDC), and North China (NC). The phenotypes of indigenous pig breeds have large differences among the different regions. Furthermore, pigs in the southern region and plateau are smaller than the northern pigs, which is partly related to their environment and climate [8,9]. The climate of Northern China is cold and dry, and Southern China has a hot and humid climate [10]. Moreover, the environment is the main factor affecting the distribution and diversity of species [8,11,12]. Animals can become better adapted to their local environments through genetic variation associated with environmental tolerances [8,13]. Pig breeds in the southern region are well adapted to the heat and humidity [8], and Tibetan pigs are able to adapt to the harsh environment of high altitudes and hypobaric and hypoxic conditions in the plateau [14]. Indeed, the indigenous pigs in different geographical areas have evolved adaptations to their region producing distinct representative characteristics. 

In this study, a population genetic analysis was performed on six geographically isolated Chinese indigenous pig meta-populations. Genetic and allelic diversity of each indigenous pig meta-population was evaluated to determine their proportional contributions to the gene pool of all six meta-populations. Finally, the signatures of selective sweeps through Chinese indigenous pig meta-populations were compared to screen potential candidate genes associated with acclimation. 

## 2. Material and Methods

### 2.1. Sample Collection

**Figure 1:**
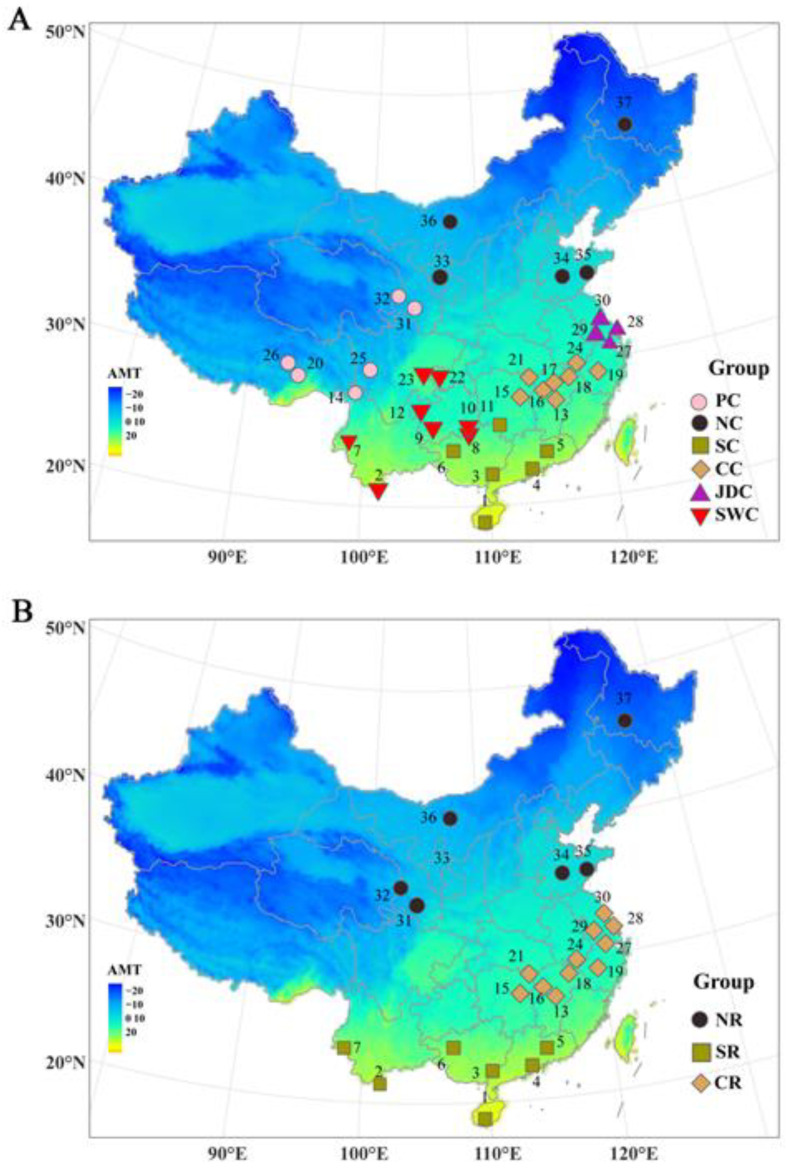
Geographical distributions of 37 Chinese indigenous pig breeds. Thirty-seven populations of Chinese indigenous pigs: 1, Wuzhishan pig; 2, Diannanxiaoer pig; 3, Luchuan pig; 4, Guangdongdahuabai pig; 5, Lantang pig; 6, Bamaxiang pig; 7, Mingguangxiaoer pig; 8, Congjiangxiang pig; 9, Guanling pig; 10, Xiang pig; 11, Dongshan pig; 12, Kele pig; 13, Leanhua pig; 14, Diqing Tibetan pig; 15, Shaziling pig; 16, Ganxiliangtouwu pig; 17, Nanchang Wild Boar pig; 18, Leping Spotted pig; 19, Jinhua pig; 20, Milin Tibetan pig; 21, Tongcheng pig; 22, Rongchang pig; 23, Neijiang pig; 24, Wannan Spotted pig; 25, Litang Tibetan pig; 26, Gongbujiangda Tibetan pig; 27, Sutai pig; 28, Meishan pig; 29, Erhualian pig; 30, Jiangquhai pig; 31, Tibetan pig; 32, Gansu Tibetan pig; 33, Bamei pig; 34, Laiwuhei pig; 35, Lichahei pig; 36, Hetaodaer pig; and 37, Min pig. (**A**) Six geographically isolated Chinese indigenous pig meta-populations: PC, Plateau of China; NC, North China; SC, Southern China; CC, Central China; JDC, Jianghai district of China; and SWC, Southwestern China. (**B**) Three climatically isolated Chinese indigenous pig meta-populations: NR, northern region; SR, southern region; and CR, central region.

A broad array of 60K genome-wide single-nucleotide polymorphism (SNP) data that included 613 pigs from 37 Chinese indigenous breeds was downloaded [15]. Based on geographical location, we grouped these indigenous pig breeds into six meta-populations, which consisted of Southern China (SC), Southwestern China (SWC), Central China (CC), Plateau of China (PC), Jianghai district of China (JDC), and North China (NC). In addition, we also divided three climatic regions, including the northern region (NR), southern region (SR), and central region (CR), to partition Chinese indigenous pig meta-populations based on climate. The detailed distribution of these meta-populations and breeds is shown in Figure 1, and supplemental information is also provided in Table S1. 

### 2.2. Data Quality Control

Quality control of SNP data was performed using PLINK software (https://www.cog-genomics.org/plink/1.9/, accessed on 12 May 2022). The SNPs with call rates of less than 99% and minor allele frequency larger than 1% and located on sex chromosomes were removed. A set of 40,127 SNPs remained for subsequent analyses. 

### 2.3. Population Structure Analysis

The geographical distributions of the six pig meta-populations were created using ‘tmap’ functions (https://cran.r-project.org/web/packages/tmap/index.html, accessed on 12 May 2022) in R (http://www.r-project.org/, accessed on 12 May 2022). Principal component analysis (PCA) was performed using PLINK, and the visualization of PCA was created with ggplot2 (http://had.co.nz/ggplot2/, accessed on 12 May 2022). The phylogenetic tree was analyzed with MEGA6 [16] and drawn by using ggtree (http://www.bioconductor.org/packages/ggtree, accessed on 12 May 2022). ADMIXTURE software [17] was utilized to reveal admixture patterns among the six pig meta-populations. Pophelper (http://www.royfrancis.com/pophelper/, accessed on 12 May 2022) was used to visualize the results of admixture patterns. The best number of ancestral components, K, was determined by cross-validation error analysis with K = 2 to 6.

### 2.4. Genetic Diversity and Differentiation

Observed heterozygosity (*H_O_*) and expected heterozygosity (*H_E_*) of the six pig meta-populations were calculated using PLINK. Metapop2 software (v2.4.2, Eugenio López-Cortegano, Spain) was used to compute the pair-wise genetic differentiation index (*F_ST_*) and pair-wise *Nei’s* minimum genetic distance (*D_nei_*) between subpopulations [18]. The total heterozygosity was partitioned into two components including the average expected heterozygosity within subpopulations (*H_S_*) and average *Nei’s* minimum genetic distance between subpopulations (*D_G_*); *H_S_* and *D_G_* were used to evaluate the contribution of each pig meta-population to heterozygosity [19]. Total allelic diversity (*A_T_*) was divided into within and between subpopulations. Average allelic diversity within subpopulations was computed as the mean number of alleles in the subpopulations minus one (*A_S_*), and the average number of special alleles in a subpopulation contrasted with the other subpopulation mean over all possible subpopulation pairs (*D_A_*) and was defined as the average allelic diversity between subpopulations [18]. 

### 2.5. Identification of Runs of Homozygosity and Distribution

We used the detectRUNS package (https://cran.r-project.org/web/packages/detectRUNS/index.html, accessed on 12 May 2022) to calculate runs of homozygosity (ROH) segments with default parameters as follows: the ROHs were divided into five categories: 0 to <6 Mb, 6 to <12 Mb, 12 to <24 Mb, 24 to <48 Mb, and ≥48 Mb. A sliding window analysis with window size of 15 SNPs was used, and the minimum number of homozygous SNPs was set to 20. Further, the homozygous threshold of windows overlapping was set to 0.05 with a minimum number of 1000 SNPs per kbps. Additionally, the maximum distance between two SNPs and the minimum length of a homozygous run was set to 1,000,000 bps and 250,000 bps, respectively. The individual genomic inbreeding coefficient based on ROH (*F_ROH_*) [20] of the six geographically isolated meta-populations was also calculated using detectRUNS. The results of inbreeding coefficients for different meta-populations were plotted by ggplot2.

### 2.6. Selective Sweep Analysis 

The population differentiation index (*F_ST_*) and cross-population composite likelihood ratio test (*XP-CLR*) were employed to detect the genomic regions under selection for the differentiation of Chinese indigenous pigs. Selective sweep analysis was performed using VCFtools [21] and XPCLR software [22]. Manhattan maps of the *F_ST_* and *XP-CLR* results were plotted using rMVP (https://github.com/xiaolei-lab/rMVP, accessed on 12 May 2022). Selective sweeps were scanned for *F_ST_* and *XP-CLR* using a 10 kb non-overlapping window. Genomic signatures were defined as the top 1% of *F_ST_* and *XP-CLR*. In addition, the intersection of genes located around the significant selection regions were annotated using BioMart (http://asia.ensembl.org/biomart/martview/, accessed on 12 May 2022).

## 3. Results and Discussion

### 3.1. Population Structure

**Figure 2:**
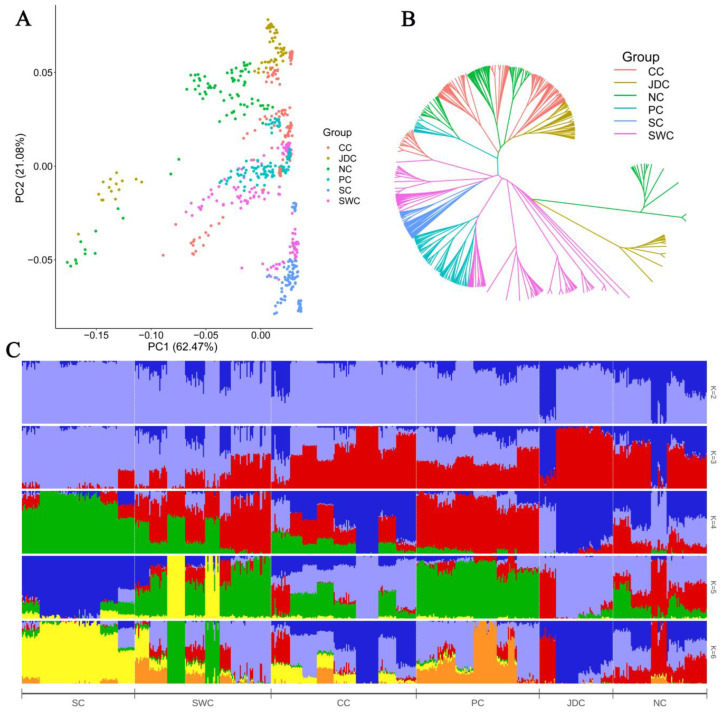
Population structures and relationships. (**A**) Principal component analysis of Chinese indigenous pig populations. (**B**) Phylogenetic tree of Chinese indigenous pig populations. Group colors used in (**A**) are the same as (**B**). (**C**) Population structure of Chinese indigenous pigs revealed by admixture analysis. The population abbreviations are as in Figure 1A.

According to their geographic origin and characteristics, Chinese indigenous pigs were divided into six geographically isolated meta-populations (Figure 1A) [23,24,25,26]. Principal component analysis based on whole-genome-wide SNPs revealed that the six meta-populations were relatively mixed (Figure 2A). Principal component 1 (PC1) captured 62.47% of the variance and separated some individuals in the Jianghai district of China (JDC) and North China (NC) from the main cluster. Interestingly, PC2 arranged the six meta-populations into groups somewhat consistent with their geographic origin with the Southern China (SC) and Southwestern China (SWC) meta-populations having maximum negative values and the NC and JDC meta-populations having maximum positive values (Figure 2A). Moreover, clear population admixture was seen in phylogenetic clustering (Figure 2B) and population structure (Figure 2C). A possible reason for this result is that the occupation of adjacent regions facilitates gene flow among these pig breeds. A population structure analysis provided evidence for introgression from exotic ancestry into Chinese indigenous pigs (Figure 2C), consistent with previous studies [27]. In addition, there were more genetic components in the SWC and CC meta-populations, which was consistent with the results of a frequent gene exchange in southern China [28]. The SC meta-population had unique genetic features compared to the others (Figure 2B,C), which could result from unique selective pressures from the hot and humid climate and from artificial selection for a small body size by humans [29] or long-term ancestral isolation. 

### 3.2. Genetic Diversity and Differentiation

**Figure 3:**
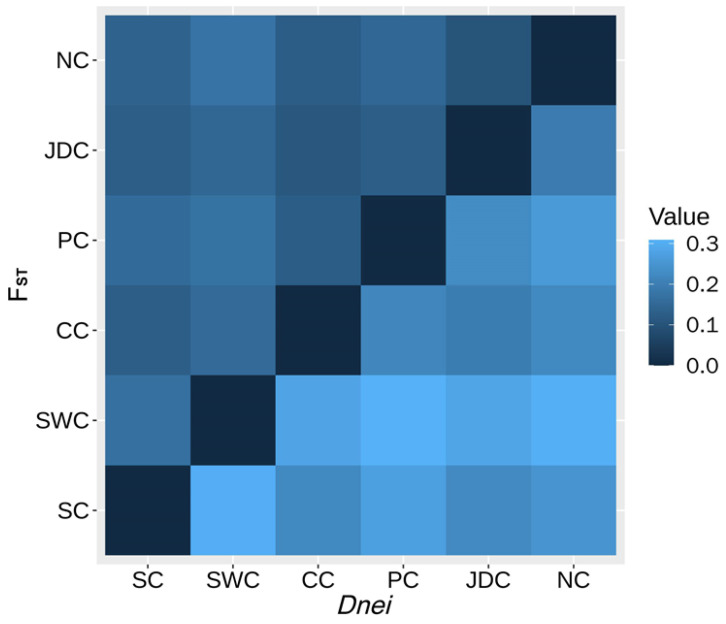
Heatmap plot of *Nei’s* minimum genetic distance *Dnei* (lower) and gene frequency differentiation index *F_ST_* (upper). The population abbreviations are as in Figure 1A.

Genetic diversity was calculated for each pig meta-population based on the SNP data (Table 1). The highest observed heterozygosity (*H_O_*: 0.2790) and expected heterozygosity (*H_E_*: 0.3224) were found for the NC meta-population; this meta-population had the lowest inbreeding coefficient (*F*: 0.3533) and average co-ancestry (*f_ii_*: 0.6817). The minimum *H_O_* (0.2246) and *H_E_* (0.2733) were seen for the CC (Central China) and PC (Plateau of China) meta-populations, respectively. The maximum *F* (0.4748) and *f _ii_* (0.7676) were observed in the JDC and SC meta-populations, respectively (Table 1). The *F_ST_* and *D_nei_* results between the six pig meta-populations are shown in Figure 3. The values of *F_ST_* and *D_nei_* between the SWC (Southwestern China) and PC meta-populations were the lowest among all pair-wise comparisons of the meta-populations with values of 0.0375 and 0.019, respectively. In addition, the SC and JDC meta-populations had the highest values of differentiation indices (*F_ST_*: 0.1359 and *D_nei_*: 0.0709). The values for genetic differences were largely consistent with the physical distances between geographical coordinates, indicating that geographical distance influences the degree of genetic differentiation. Comparing the CC meta-population with the other meta-populations produced the lowest averages of *F_ST_* (0.06486) and *D_nei_* (0.03254). This finding of similar genetic composition of the CC meta-population to the others may be the result of its location in the middle region of China leading to a high gene flow between the CC meta-population and the others. Similarly, the results for population structure indicate that the CC meta-population was admixed with other populations (Figure 2C). The highest averages of *F_ST_* and *D_nei_* were between the SC meta-population and the other populations with values of 0.10126 and 0.05288, respectively, which may be from the adaptation to its unique geographic location and relative isolation. 

### 3.3. Contribution to Genetic and Allelic Diversity

**Figure 4:**
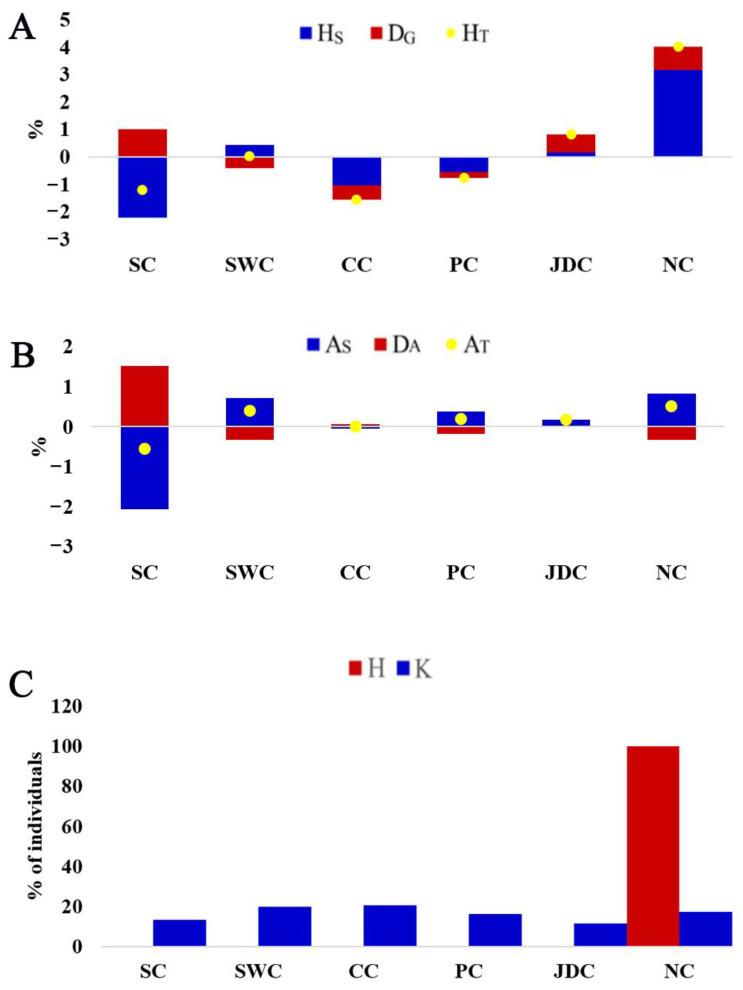
Contribution to genetic diversity of 6 Chinese indigenous pig meta-populations. (**A**,**B**) Contribution to within-(*H_S_*, *A_S_*), between-(D_G_, D_A_), and total (*H_T_*, *A_T_*) genetic and allelic diversity of six Chinese indigenous pig meta-populations. (**C**) Contribution of individuals (%) from each meta-population to a synthetic pool of individuals with maximal genetic diversity (*H*) or number of alleles (*K*). The population abbreviations are as in Figure 1A.

Maintenance of maximum genetic diversity is an important goal of conservation to maintain the potential for adaptation to environmental changes in the future [30]. Expected heterozygosity and allelic diversity measure genetic diversity, and they are susceptible to selection intensity and the influence of bottlenecks, respectively [31]. The contribution of each meta-population to the global genetic diversity was determined by calculating the change in genetic and allelic diversity, removing one subpopulation from all meta-populations before the calculation. When a subpopulation is removed, the positive and negative contribution values represent the loss and gain of diversity, respectively [18]. These positive and negative values can be used to rank the conservation priority of the subpopulation. Conservation priority based on contribution rank to total diversity within the meta-populations was similar for total heterozygosity and allelic diversity (Figure 4). The removal of the NC meta-population resulted in the highest loss of total heterozygosity (*H_T_*, 4.0108%) followed by the JDC meta-population (*H_T_*, 0.8306%) (Figure 4A). Furthermore, the NC and SWC meta-populations had the first-(0.5118%) and second-(0.3959%) highest contribution to the loss of total allelic diversity (*A_T_*), respectively. The CC meta-population had the highest percentage of *H_T_* (−1.5417%), and the percentage of *A_T_* was the highest in the SC meta-population (−0.5499%). The SC meta-population had lower within-population diversity because it had the most significant negative contributions to both *H_S_* (−2.1954%) and *A_S_* (−2.0666%) (Figure 4A,B). Moreover, the SC meta-population had the highest average co-ancestry (*f_ii_*: 0.7676), which corresponds to the above results for population diversity (Table 1). The NC meta-population had the largest within-population diversity based on the highest values of positive contribution to both *H_S_* (3.1711%) and *A_S_* (0.8303%). Additionally, the largest within-population diversity corresponded to a low average co-ancestry (*f_ii_*: 0.6817) in the NC meta-population (Table 1). The maximum value of expected heterozygosity roughly corresponded to the minimum value of average co-ancestry [32]. However, the lowest negative contribution to *D_G_* (−0.4937%) was observed in the CC meta-population, which is consistent with the CC meta-population having the smallest average *Nei’s* distance compared with the others (*D_nei_*: 0.02712) (Figure 3). Based on maximum expected heterozygosity and total number of alleles, we calculated the contribution of each meta-population to the total meta-population gene pool (Figure 4C). As expected, the NC population had the highest contribution to allelic diversity (*K*). For genetic diversity, the NC population contributed 100%, and all six meta-populations contributed greater than 10% to allelic diversity. Overall, the above evidence suggests that the NC meta-population should be given the highest priority in a conservation plan for Chinese indigenous pigs. We should strengthen the research and application of indigenous pigs in North China, promote the re-innovation of pig germplasms, and speed up the development of local pig industries. 

### 3.4. Distribution of ROH and Genomic Inbreeding Coefficient

**Figure 5:**
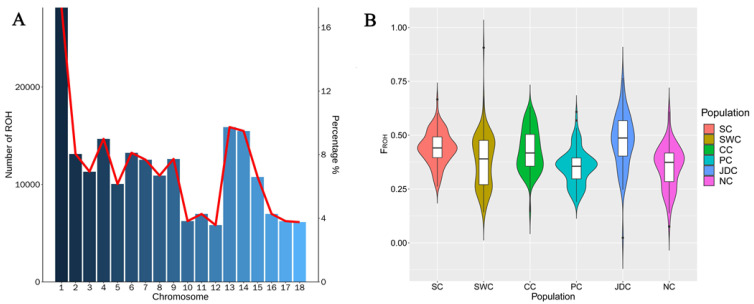
Descriptive graphics of runs of homozygosity (ROH) in 6 geographically isolated Chinese indigenous pig meta-populations. (**A**) The average number of ROHs per chromosome (bars) and the average percentage of each chromosome covered by ROHs (lines) for all pigs. (**B**) Distribution of ROH-based inbreeding coefficients (*F_ROH_*) on each pig population within geographic region. The population abbreviations are as in Figure 1A.

A total of 207,141 ROHs were identified in the 613 pigs by detectRUNS, and the mean length for each ROH was 2.91 Mb. The number of ROHs on each pig chromosome is displayed in Figure 5A. Figure 5A illustrates that the number of ROH fragments may be related to chromosome length for each chromosome. The individual genomic inbreeding coefficient (*F_ROH_*) for the pig meta-populations within geographic regions is shown in Figure 5B. The NC (0.3533) and PC (0.3536) meta-populations had a lower average inbreeding coefficient due to their wide distribution, indicating the possible influence of geographical distribution on inbreeding coefficients. The JDC meta-population had the highest average inbreeding coefficient (Table 1, 0.4748), but the number of ROH segments in this population was the lowest (Table 2). These results indicate that this population had a greater number of long ROH segments and had recently experienced higher inbreeding than the other meta-populations. This finding may be related to the close physical distance of breeds in the JDC meta-population. These results may indicate that reducing inbreeding among species and improving population diversity can be achieved by increasing the physical distance between species. 

### 3.5. Selective Sweep Analysis for Temperature Adaptation

**Figure 6:**
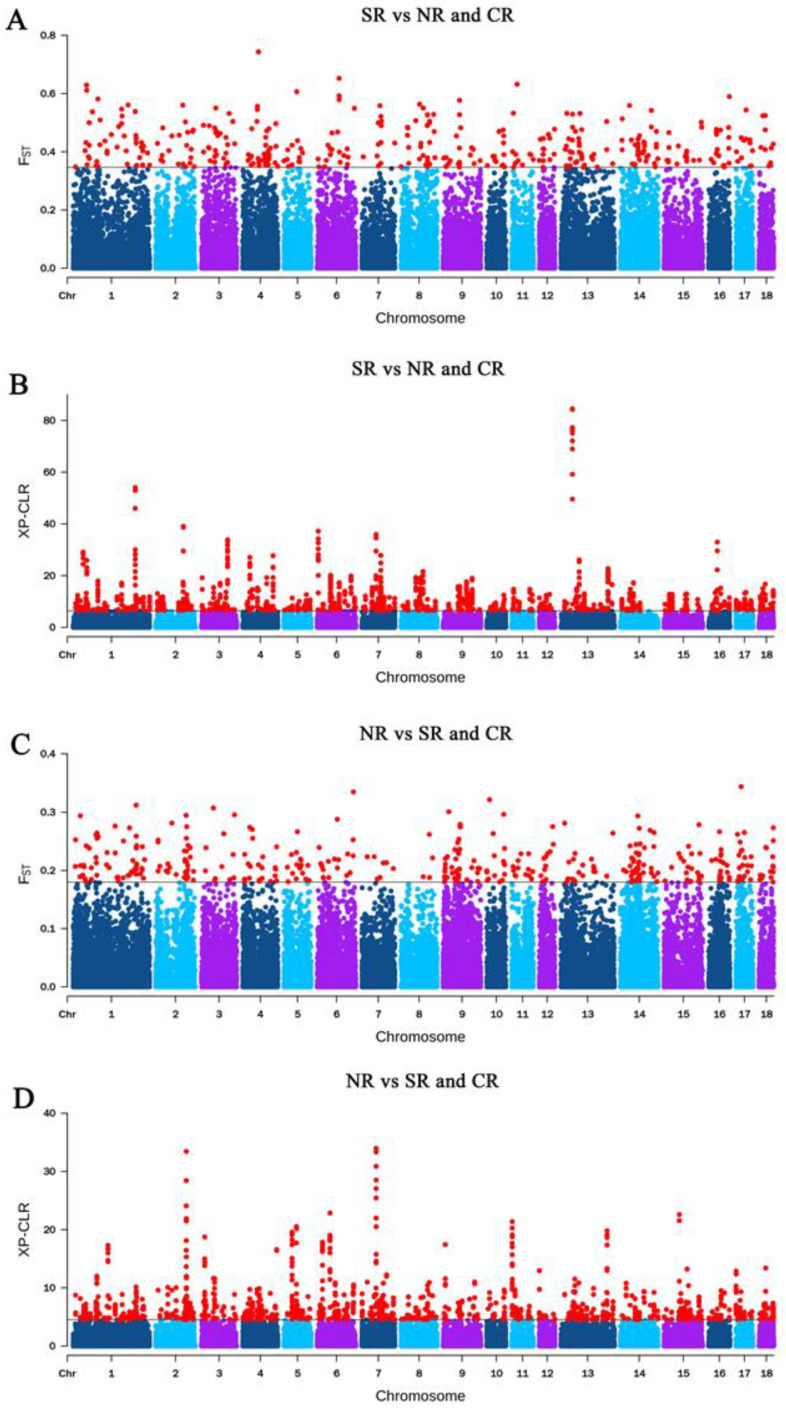
Selective sweep analysis for three climatically isolated Chinese indigenous pig meta-populations by using *F_ST_* and *XP-CLR* (Manhattan map of *F_ST_* and Manhattan map of *XP-CLR*.). The red dots indicate the top 1% values of *F_ST_* and *XP-CLR*. (**A**) *F_ST_* of SR vs. NR and CR. (**B**) *XP-CLR* of SR vs. NR and CR. (**C**) *F_ST_* of NR vs. SR and CR. (**D**) *XP-CLR* of NR vs. SR and CR. The population abbreviations are as in Figure 1B.

Chinese indigenous pigs were domesticated from wild ancestors and then differentiated into various breeds. To adapt to the local climate and environment, unique genetic structures developed over the past several thousand years. The annual mean temperature (AMT) in southern China is over 20 °C, whereas the AMT in northern China is below −20 °C (Figure 1B). Pigs residing in the southern region (SR) adapted to heat, while pigs living in the northern region (NR) adapted to cold. Thus, three populations comprised of breeds from the southern region (SR), central region (CR), and northern region (NR) were compared (Figure 6). In addition, Figure 2C shows that the consanguineous composition of pig breeds in SR (consisting mainly of SC and SWC), CR (consisting mainly of JDC and CC), and NR (consisting of NC) is obviously different. The consanguineous composition of CR was more like one combination of SR and CR. Using SR vs. NR and CR (SNC) and NR vs. SR and CR (NSC), we were more likely to detect the true difference of environmental adaptability between the northern and southern regions. Two combinations (Figure 6A,B and Figure 6C,D), SR vs. NR and CR (*F_ST_* > 0.3430, *XP-CLR* > 6.1482) and NR vs. SR and CR (*F_ST_* > 0.1787, *XP-CLR* > 4.3531), were used to explore the selection signatures related to heat and environmental adaptability, respectively (Figure 1B). Genes located within the 10 kb region adjacent to the selective region were defined as potential candidate genes. The overlap genes of *F_ST_* and *XP-CLR* were 59 and 87 in SNC and NRC, respectively. A total of six candidate genes were identified underling environmental adaptation, possibly through changes in heat response and fat deposition (Table 3). 

Genes associated with heat stress response included *EPAS1*, *NFE2L2*, and *VPS13A*. *EPAS1* is associated with heat tolerance and can be directly implicated in the hypoxic response during thermal stress [33,34,35]. This finding suggests that *EPAS1* may protect the reproductive capacity of pigs. A previous study indicated that *NFE2L2* plays a protective role in bovine endometrial epithelial cells cultured under heat stress conditions [36]. *NFE2L2* may reduce the effects of heat on fertility in pigs in the southern region. In addition, *VPS13A* has been recognized as a key regulator of secretion and aggregation of blood platelets in heat stress conditions, which can reduce physiological damage, including the risk of coronary thrombosis, in animals under heat stress [37]. This finding suggests that *VPS13A* is likely to have a close relationship with heat stress resistance in pigs. These three genes possibly reduced or mitigated inflammatory responses produced by heat stress in local pigs, and they may be associated with high-temperature adaptation of pigs in southern China. 

Three genes associated with fat deposition were *SPRY1*, *PLA2G4A*, and *UBE3D*. Adipose tissue-specific *SPRY1* expression in mice resulted in increased body fat, and conditional deletion of *SPRY1* resulted in increased body fat [38]. Furthermore, some studies demonstrated that *SPRY1* has protective effects on mice fed a high-energy diet, and *SPRY1* expression can prevent high-fat, diet-induced obesity [39]. This mechanism may be associated with higher lean meat of pigs in the SR. High amounts of lean meat may dissipate heat and allow for easier adaptation to high temperatures. *PLA2G4A* was a key regulator for fat deposition significantly involved in several biological processes of fat deposition [40]. Further, *PLA2G4A* was significantly associated with persistent weight stability and insulin sensitivity [41]. This result may be related to the continuous provision of energy for pigs to resist the cold. Some studies found that *UBE3D* had a relationship with fat composition in sheep, and it was a cold-resistant candidate gene in Weining cattle [42,43]. Therefore, *UBE3D* may be related to the adaptation of pigs to low temperatures in the northern region. Thick layers of subcutaneous fat are an important adaptation to cold and heat; the fat can produce heat in response to cold exposure and protect animals against hypothermia [44]. The above results indicate that the genes discussed above may play key roles in the adaptation of pigs to their environment. 

## 4. Conclusions

In summary, this study investigated contributions to genetic diversity of Chinese indigenous pig meta-populations and identified selective candidate genes involved in heat and cold environmental adaptation. We found that the NC meta-population has the highest genetic and allelic diversity for the six pig meta-populations. Numerous genes linked to fat deposition and heat stress were detected based on selective sweep analysis including *EPAS1*, *NFE2L2*, *VPS13A*, *SPRY1*, *PLA2G4A*, and *UBE3D*. These findings illuminate the genetic features of Chinese indigenous pigs leading to an improved conservation plan and provide theoretical support for the evolution of environmental adaptability.

## Figures and Tables

**Table 1:** Genetic diversity in 6 geographically isolated Chinese indigenous pig meta-populations.

Meta-Population	Origin	Breed Number	Number	*H_O_*	*H_E_*	*F*	*f_ii_*
PC	Plateau of China	6	110	0.2488	0.2733	0.3536	0.7428
NC	North China	5	84	0.2790	0.3224	0.3533	0.6817
SC	Southern China	6	101	0.2525	0.2953	0.4407	0.7676
CC	Central China	8	130	0.2246	0.2752	0.4278	0.7470
JDC	Jianghai District of China	4	66	0.2289	0.2882	0.4748	0.7318
SWC	Southwestern China	8	122	0.2415	0.2773	0.3823	0.7308

Note: *H_O_*, observed heterozygosity; *H_E_*, expected heterozygosity; *F*, inbreeding coefficient; and *f_ii_*, average co-ancestry within the population.

**Table 2:** Descriptive statistics of runs of homozygosity (ROH) number of each pig population within geographic region by ROH length class (0–6 Mb, 6–12 Mb, 12–24 Mb, 24–48 Mb, and >48 Mb). The population abbreviations are as in Figure 1A.

Class	0–6 Mb	6–12 Mb	12–24 Mb	24–48 Mb	>48 Mb	Total
PC	35,759 (95.07%)	1265 (3.36%)	372 (0.99%)	142 (0.38%)	74 (0.20%)	37,612
NC	21,126 (91.63%)	1204 (5.22%)	438 (1.90%)	197 (0.85%)	92 (0.40%)	23,057
SC	36,375 (93.72%)	1713 (4.41%)	467 (1.20%)	152 (0.39%)	107 (0.28%)	38,814
CC	41,530 (92.92%)	1945 (4.35%)	703 (1.57%)	326 (0.73%)	189 (0.42%)	44,693
JDC	20,526 (90.18%)	1376 (6.05%)	541 (2.38%)	237 (1.04%)	81 (0.36%)	22,761
SWC	37,518 (93.32%)	1731 (4.31%)	622 (1.55%)	231 (0.57%)	102 (0.25%)	40,204

**Table 3:** List of candidate genes located in genomic regions with a high value of both the intersection of *F_ST_* and *XP-CLR*. The population abbreviations are as in Figure 1B.

Group	CHR	Start (bp)	End (bp)	Gene	*F_ST_*	*XP-CLR*	Function
SNC	3	94,167,759	94,253,662	*EPAS1*	0.4493	13.6013	Heat tolerance in dairy cows [33,34,35]
SNC	15	82,967,485	83,146,185	*NFE2L2*	0.3681	7.8295	Protecting the body of dairy cows under heat stress [36]
SNC	1	230,069,339	230,331,343	*VPS13A*	0.4295	8.3043	Reducing physiological damage in animals under heat stress [37]
SNC	8	100,449,476	100,863,780	*SPRY1*	0.4900	6.9364	Fat deposition [38,39]
NSC	9	127,853,581	128,164,825	*PLA2G4A*	0.1960	5.0068	The key regulators for fat deposition [40,41]
NSC	1	83,140,479	83,295,865	*UBE3D*	0.2612	7.2031	Related to fat composition in sheep [42,43,44]

Note: SNC, SR vs. NR and CR; NSC, NR vs. SR and CR.

## Data Availability

The data used in this study are publicly available, and can be obtained from: http://dx.doi.org/10.5061/dryad.30tk6 (accessed on 5 May 2021).

## Supplementary Materials

The following supporting information can be downloaded at: https://www.mdpi.com/article/10.3390/ani13081396/s1, Table S1: Breeds information.
